# Probabilistic Ensemble of Deep Information Networks

**DOI:** 10.3390/e22010100

**Published:** 2020-01-14

**Authors:** Giulio Franzese, Monica Visintin

**Affiliations:** Electronic and Telecommunications, Politecnico di Torino, 10100 Torino, Italy; monica.visintin@polito.it

**Keywords:** information theory, information bottleneck, classifier, decision tree, ensemble

## Abstract

We describe a classifier made of an ensemble of decision trees, designed using information theory concepts. In contrast to algorithms C4.5 or ID3, the tree is built from the leaves instead of the root. Each tree is made of nodes trained independently of the others, to minimize a local cost function (information bottleneck). The trained tree outputs the estimated probabilities of the classes given the input datum, and the outputs of many trees are combined to decide the class. We show that the system is able to provide results comparable to those of the tree classifier in terms of accuracy, while it shows many advantages in terms of modularity, reduced complexity, and memory requirements.

## 1. Introduction

Supervised classification is at the core of many modern applications of machine learning. The history of classifiers is rich and many variants have been proposed, such as decision trees, logistic regression, Bayesian networks, and neural networks (for an overview of general methods, see [1,2,3]). Despite the power of modern deep learning, for many problems involving categorical structured datasets, decision trees [4,5,6,7] or Bayesian networks [8,9,10] usually outperform neural network based approaches.

Decision trees are particularly interesting because they can be easily interpreted. Various types of tree classifiers can be discriminated according to the metric for the iterative construction and selection of features [4]: popular tree classifiers are based on information theoretic metrics, such as ID3 and C4.5 [6,7]. However, it is known that the greedy splitting procedure at each node can be sub-optimal [11], and that decision trees are prone to overfitting when dealing with small datasets. When a classifier is not strong enough, there are, roughly speaking, two possibilities: choosing a more sophisticated classifier or ensembling multiple “weak” classifiers [12,13]. This second approach is usually called the *ensemble* method. In the performance tradeoff by using multiple classifiers simultaneously, we improve classification performance, paying with the loss of interpretability.

The so-called “information bottleneck”, described by Tishby and Zaslavsky [14] and Tishby et al. [15], was proposed in [16] to build a classifier (Deep Information Network, DIN) with a tree topology that compresses the input data and generates the estimated class. DINs [16] are based on the so-called information node that, using the input samples of a feature $X_{in}$, generates samples of a new feature $X_{out}$, according to the conditional probabilities $P(X_{out}=j|X_{in}=i)$ obtained by minimizing the mutual information $I(X_{in};X_{out})$, with the constraint of a given mutual information $I(X_{out};Y)$ between $X_{out}$ and the target/class *Y* (information bottleneck [14]). The outputs of two or more nodes are combined, without information loss, to generate samples of a new feature passed to a subsequent information node. The final node (root) directly outputs the class of each input datum. The tree structure of the network is thus built from the leaves, whereas C4.5 and ID3 build it from the root.

We here propose an improved implementation of the DIN scheme in [16] that only requires the propagation through the tree of small matrices containing conditional probabilities. Notice that the previous version of the DIN was stochastic, while the one we propose here is deterministic. Moreover, we use an ensemble (e.g., [12,13]) of trees with randomly permuted features and weigh their outputs to improve classification accuracy.

The proposed architecture has several advantages in terms of:

- extreme flexibility and high modularity: all the nodes are functionally equivalent and with a reduced number of inputs and outputs, which gives good opportunities for a possible hardware implementation;
- high parallelizability: each tree can be trained in parallel with the others;
- memory usage: we need to feed the network with data only at the first layer and simple incremental counters can be used to estimate the initial probability mass distribution; and
- training time and training complexity: the locality of the computed cost function allows a nodewise training that does not require any kind of information from other points of the tree apart from its feeding nodes (that are usually a very small number, e.g., 2–3).

With respect to the DINs in [16], the main difference is that samples of the random variables in the inner layers of the tree are never generated, which is an advantage in the case of large datasets. However, an assumption of statistical independence (see Section 2.3) is necessary to build the probability matrices and this might be seen as a limitation of the newly proposed method. However, experimental results (see Section 5) show that this approximation does not compromise the performance.

We underline similarities and differences of the proposed classifier with respect to the methods described in [6,7] since they are among the best performing ones. When using decision trees, as well as DINs, categorical and missing data are easily managed, but continuous random variables are not: quantization of these input features is necessary in a pre-processing phase, and it can be performed as in C4.5 [6], using other heuristics, or manually. Concerning differences, instead, the first one is that normally a hierarchical decision tree is built starting from the root and splitting at each node, whereas we here propose a way to build a tree starting from the leaves. The topology of our network implies that, once the initial ordering of the features has been set, there is no need, after each node is trained, to perform a search of the best possible next node. The second important difference is that we do not use directly mutual information as a metric for building the tree but we base our algorithm on the Information Bottleneck principle [14,15,17,18,19,20,21]. This allows us to extract all the relevant information (the *sufficient statistic*) while removing the redundant one, which is helpful in avoiding overfitting. As in [12,13], we use an ensemble method. We choose the simplest possible form of ensemble combination: we train independently many structurally equivalent networks, using the same single dataset but permuting the order of the features, and produce a weighted average of the outputs based on a simple rule described in Section 3.1. Notice that we use a one-shot procedure, i.e., we do not iterate more than once over the entire dataset and exploit techniques similarly to [22,23]. We leave the study of more sophisticated techniques to future works.

Section 2 and Section 3 more precisely describe the structure of the DIN and how it works, Section 4 gives some insight on the theoretical properties, Section 5 comments the results obtained with standard datasets. Conclusions are finally drawn in Section 6.

## 2. The DIN Architecture and Its Training

The information network is made of input nodes (Section 2.1), information nodes (Section 2.2), and combiners joined together through a tree network described in Section 2.3. Moreover, an ensemble of $N_{mach}$ trees is built, based on which the final estimated class is produced (Section 3.1). In [16], the input nodes are not present, the information node has a slightly different role, the combiners are much simpler than those described here, and just one tree was considered. As already stated, the new version of the DIN is more efficient when a large dataset with relatively few features is analyzed.

In the following, it is assumed that all the features take a finite number of discrete values; a case of continuous random variables is discussed in Section 5.2.

It is also assumed that $N_{train}$ points are used in the training phase, $N_{test}$ points in the testing phase, and that *D* features are present. The *n*th training point corresponds to one of $N_{class}$ possible classes.

### 2.1. The Input Node

**Figure 1:**
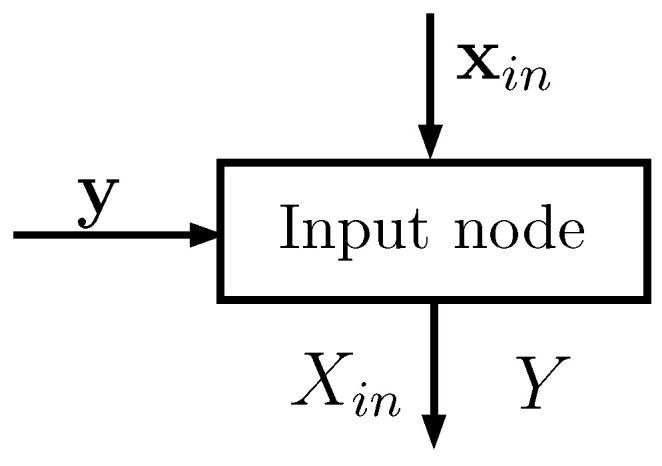
Schematic representation of an input node: the inputs are two vectors and the outputs are matrices that statistically describe the random variables $X_{in}$ and *Y*.

Each input node (see Figure 1) has two input vectors:

1. $x_{in}$ of size $N_{train}$, whose elements take values in a set of cardinality $N_{in}$; $x_{in}$ corresponds to one of the *D* features of the dataset (typically one column)
2. y of size $N_{train}$, whose elements take values in a set of cardinality $N_{class}$; y corresponds to the known classes of the $N_{train}$ points

The notation we use in the equations below is the following: $Y,X_{in}$ represent random variables; y(n) and $x_{in}\left(n\right)$ are the *n*th elements of vectors y and $x_{in}$, respectively; and 1(c) is equal to 1 if *c* is true, and is otherwise equal to 0. Using Laplace smoothing [2], the input node estimates the following probabilities (the probability mass function of *Y* in Equation (1) is common to all the input nodes: it can be evaluated only by the first one and passed to the others):

(1) $$\hat{P}\left(Y=m\right)≃\frac{1+\sum_{n=0}^{N_{train}-1}1\left(y\left(n\right)=m\right)}{N_{train}+N_{class}}\;\;m=0,\ldots ,N_{class}-1$$

(2) $$\hat{P}\left(X_{in}=i\right)≃\frac{1+\sum_{n=0}^{N_{train}-1}1\left(x_{in}\left(n\right)=i\right)}{N_{train}+N_{in}},\;i=0,\ldots ,N_{in}-1$$

(3) $$\begin{array}{c} \hat{P}\left(Y=m,X_{in}=i\right)≃\frac{1+\sum_{n=0}^{N_{train}-1}1\left(y\left(n\right)=m\right)1\left(x_{in}\left(n\right)=i\right)}{N_{train}+N_{class}N_{in}} \end{array}$$

From basic application of probability rules, $\hat{P}\left(Y=m|X_{in}=i\right)$ and $\hat{P}\left(X_{in}=i|Y=m\right)$ are then computed. From now on, for simplicity, we denote all the estimated probabilities $\hat{P}$ simply as *P*.

All the above probabilities can be organized in matrices defined as follows:

(4) $$P_{Y}\in R^{1\times N_{class}},\;P_{Y}\left(m\right)=P\left(Y=m\right)$$

(5) $$P_{X_{in}}\in R^{1\times N_{in}},\;P_{X_{in}}\left(i\right)=P\left(X_{in}=i\right)$$

(6) $$P_{X_{in}|Y}\in R^{N_{class}\times N_{in}},\;P_{X_{in}|Y}\left(m,i\right)=P\left(X_{in}=i|Y=m\right)$$

(7) $$P_{Y|X_{in}}\in R^{N_{in}\times N_{class}},\;P_{Y|X_{in}}\left(i,m\right)=P\left(Y=m|X_{in}=i\right)$$

Note that vectors $x_{in}$ and y are not needed by the subsequent elements in the tree; only the input nodes have access to them.

Notice also that the following equalities hold:

(8) $$P_{X_{in}}=P_{Y}P_{X_{in}|Y}$$

(9) $$P_{Y}=P_{X_{in}}P_{Y|X_{in}}$$

### 2.2. The Information Node

**Figure 2:**
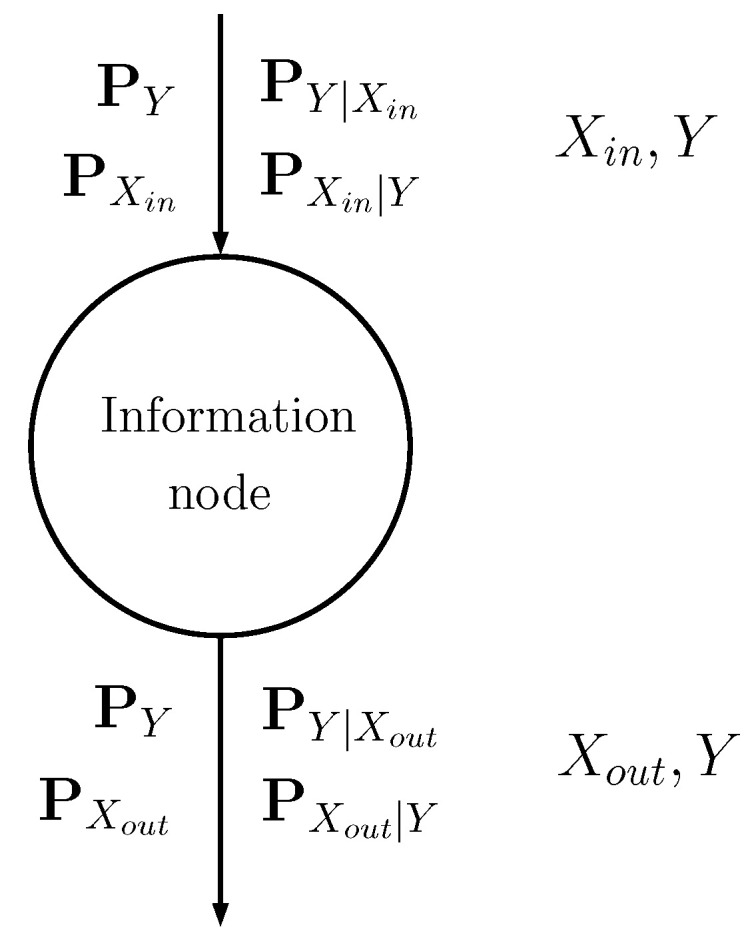
Schematic representation of an information node, showing the input and output matrices.

The information node is schematically shown in Figure 2: the input discrete random variable $X_{in}$ is stochastically mapped into another discrete random variable $X_{out}$ (see [16] for further details) through probability matrices:

- The input probability matrices $P_{X_{in}},P_{X_{in}|Y},P_{Y|X_{in}},P_{Y}$ describe the input random variable $X_{in}$, with $N_{in}$ possible values, and its relationship with class *Y*.
- The output matrices $P_{X_{out}},P_{X_{out}|Y},P_{Y|X_{out}},P_{Y}$ describe the output random variable $X_{out}$, with $N_{out}$ possible values, and its relationship with *Y*.

Compression (source encoding) is obtained by setting $N_{out}<N_{in}$.

In the training phase, the information node generates the conditional probability mass function that satisfies the following equation (see [14]):

(10) $$P\left(X_{out}=j|X_{in}=i\right)=\frac{1}{Z(i;\beta )}P\left(X_{out}=j\right)e^{-\beta d(i,j)},\;i=0,\ldots ,N_{in}-1,j=0,\ldots ,N_{out}-1$$

where

- $P(X_{out}=j)$ is the probability mass function of the output random variable $X_{out}$
  (11) $$P\left(X_{out}=j\right)=\sum_{i=0}^{N_{in}-1}P\left(X_{in}=i\right)P\left(X_{out}=j|X_{in}=i\right),\;j=0,\ldots ,N_{out}-1$$
- d(i,j) is the Kullback–Leibler divergence
  (12) $$\begin{array}{cc} d(i,j) & =\sum_{m=0}^{N_{class}-1}P\left(Y=m|X_{in}=i\right)\log_{2}\frac{P(Y=m|X_{in}=i)}{P(Y=m|X_{out}=j)}\\ \; & =\mathrm{KL}(P\left(Y|X_{in}=i\right)||P\left(Y|X_{out}=j\right)) \end{array}$$
  and
  (13) $$\begin{array}{cc} P\left(Y=m|X_{out}=j\right)=\sum_{i=0}^{N_{in}-1} & P\left(Y=m|X_{in}=i\right)P\left(X_{in}=i|X_{out}=j\right),\\ \; & m=0,\ldots ,N_{class}-1,j=0,\ldots ,N_{out}-1 \end{array}$$
- β is a real positive parameter.
- Z(i;β) is a normalizing coefficient to get
  (14) $$\sum_{j=1}^{N_{out}-1}P\left(X_{out}=j|X_{in}=i\right)=1.$$

The probabilities $P(X_{out}=j|X_{in}=i)$ can be iteratively found using the Blahut–Arimoto algorithm [14,24,25].

Equation (10) solves the information bottleneck: it minimizes the mutual information $I(X_{in};X_{out})$ under the constraint of a given mutual information $I(Y;X_{out})$. In particular, Equation (10) is the solution of the minimization of the Lagrangian

(15) $$L=I\left(X_{in};X_{out}\right)-\beta I\left(Y;X_{out}\right).$$

If the Lagrangian multiplier β is increased, then the constraint is privileged and the information node tends to maximize the mutual information between its output $X_{out}$ and the class *Y*; if β is reduced, then minimization of $I(X_{in};X_{out})$ is obtained (compression). The information node must actually balance compression from $X_{in}$ to $X_{out}$ and propagation of the information about *Y*. In our implementation, the compression is also imposed by the fact that the cardinality of the output alphabet $N_{out}$ is smaller than that of the input alphabet $N_{in}$.

The role of the information node is thus that of finding the conditional probability matrices

(16) $$P_{X_{out}|X_{in}}\in R^{N_{in}\times N_{out}},\;P_{X_{out}|X_{in}}\left(i,j\right)=P\left(X_{out}=j|X_{in}=i\right)$$

(17) $$P_{Y|X_{out}}\in R^{N_{out}\times N_{class}},\;P_{Y|X_{out}}\left(j,m\right)=P\left(Y=m|X_{out}=j\right)$$

(18) $$P_{X_{out}}\in R^{1\times N_{out}},\;P_{X_{out}}\left(j\right)=P\left(X_{out}=j\right)$$

### 2.3. The Combiner

**Figure 3:**
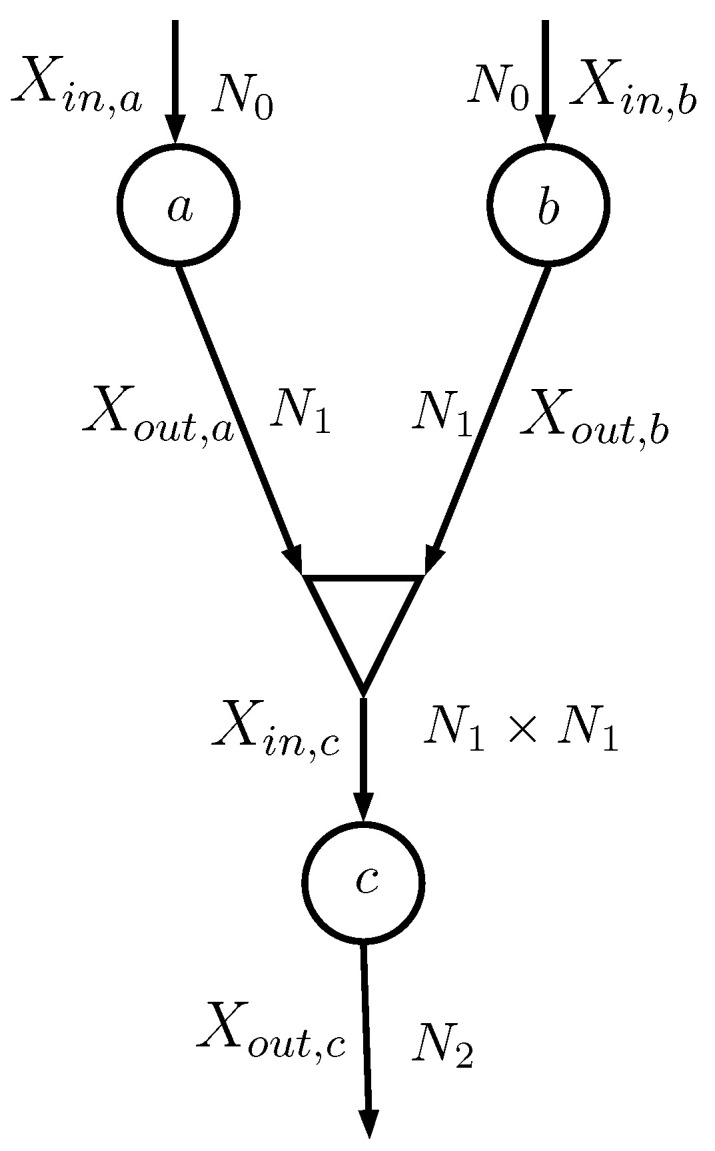
Sub-network: $X_{in,a}$, $X_{out,a}$, $X_{in,b}$, $X_{out,b}$, $X_{in,c}$, and $X_{out,c}$ are all random variables; $N_{0}$ is the number of values taken by $X_{in,a}$ and $X_{in,b}$; $N_{1}$ is the number of values taken by $X_{out,a}$ and $X_{out,b}$; and $N_{2}$ is the number of values taken by $X_{out,c}$.

Consider the case depicted in Figure 3, where the two information nodes *a* and *b* feed a combiner (shown as a triangle) that generates the input of the information node *c*. The random variables $X_{out,a}$ and $X_{out,b}$, both having alphabet with cardinality $N_{1}$, are combined together as

(19) $$X_{in,c}=X_{out,a}\;+\;N_{1}\;X_{out,b}$$

that has an alphabet with cardinality $N_{1}\times N_{1}$.

The combiner actually does not generate $X_{in,c}$; it simply evaluates the probability matrices that describe $X_{in,c}$ and *Y*. In particular, the information node *c* needs $P_{X_{in,c}|Y}$, which can be evaluated **assuming** that $X_{out,a}$ and $X_{out,b}$ are conditionally independent given *Y* (notice that in implementation [16] this assumption was not necessary):

(20) $$\begin{array}{cc} P(X_{in,c}=k|Y=m) & =P(X_{out,a}=k_{a},X_{out,b}=k_{b}|Y=m)\\ \; & =P\left(X_{out,a}=k_{a}|Y=m\right)P\left(X_{out,b}=k_{b}|Y=m\right) \end{array}$$

where $k=k_{a}+N_{1}k_{b}$. In particular, the *m*th row of $P_{X_{in,c}|Y}$ is the Kronecker product of the *m*th rows of $P_{X_{out,a}|Y}$ and $P_{X_{out,b}|Y}$

(21) $$P_{X_{in,c}|Y}\left(m,:\right)=P_{X_{out,a}|Y}\left(m,:\right)⊗P_{X_{out,b}|Y}\left(m,:\right)\;m=0,\ldots ,N_{class}-1$$

(here A(m,:) identifies the *m*th row of matrix A). The probability vector $P_{X_{in,c}}$ can be evaluated considering that

(22) $$P\left(X_{in,c}=k\right)=\sum_{m=0}^{N_{class}-1}P\left(X_{in,c}=k,Y=m\right)=\sum_{m=0}^{N_{class}-1}P\left(X_{in,c}=k|Y=m\right)P\left(Y=m\right)$$

so that

(23) $$P_{X_{in,c}}=P_{Y}P_{X_{in,c}|Y}$$

At this point, matrix $P_{Y|X_{in,c}}$ can be evaluated element by element since

(24) $$\begin{array}{cc} P(Y=m|X_{in,c}=k) & =\frac{P\left(X_{in,c}=k|Y=m\right)P\left(Y=m\right)}{P(X_{in,c}=k)},\\ \; & \;m=1,\ldots ,N_{class}-1,k=0,\ldots ,N_{1}\times N_{1}-1 \end{array}$$

It is straightforward to extend the equations to the case in which $X_{in,a}$ and $X_{in,b}$ have different cardinalities.

### 2.4. The Tree Architecture

**Figure 4:**
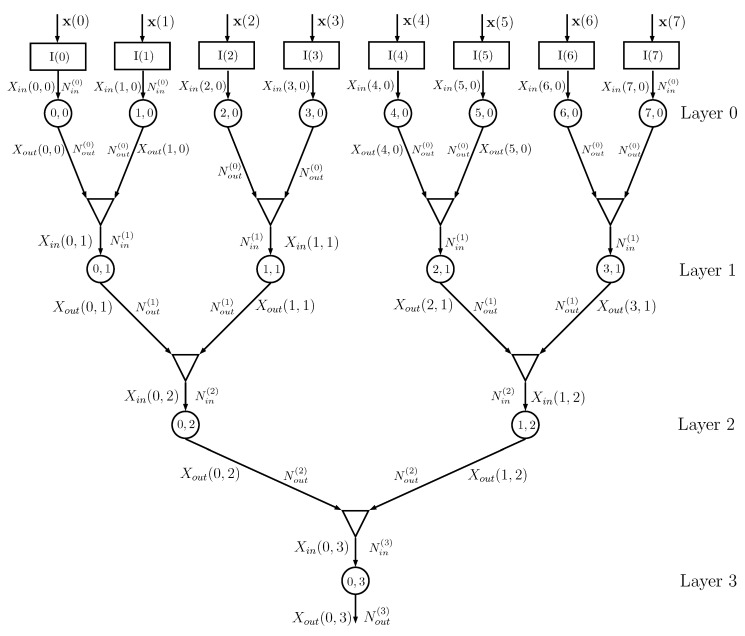
Example of a DIN for D=8: the input nodes are represented as rectangles, the info nodes as circles, and the combiners as triangles. The numbers inside each circle identify the node (position inside the layer and layer number), $N_{in}^{\left(k\right)}$ is the number of values taken by the input of the info node at layer *k*, and $N_{out}^{\left(k\right)}$ is the number of values taken by the output of the info node at layer *k*. In this example, the info nodes at a given layer all have the same input and output cardinalities.

Figure 4 shows an example of a DIN, where we assume that the dataset has D=8 features and that training is thus obtained using a matrix $X_{train}$ with $N_{train}$ rows and D=8 columns, with a corresponding class vector y. The *k*th column x(k) of matrix $X_{train}$ feeds, together with vector y, the input node I(k), k=0,…,D−1.

Information node (k,0) at layer 0 processes the probability matrices generated by the input node I(k), with $N_{in}^{\left(0\right)}$ possible values of $X_{in}\left(k,0\right)$, and evaluates the conditional probability matrices with $N_{out}^{\left(0\right)}$ possible values of $X_{out}\left(k,0\right)$, using the algorithm described in Section 2.2. The outputs of info nodes (2k,0) and (2k+1,0) are given to a combiner that outputs the probability matrices for $X_{in}\left(k,1\right)$, having alphabet of cardinality $N_{in}^{\left(1\right)}=N_{out}^{\left(0\right)}\times N_{out}^{\left(0\right)}$, using the equations described in Section 2.3. The sequence of combiners and information nodes is iterated, decreasing the number of information nodes from layer to layer, until the final root node is obtained. In the previous implementation of the DINs in [16], the root information node outputs the estimated class of the input and it is therefore necessary that the output cardinality of the root info node is equal to $N_{class}$. In the current implementation, this cardinality can be larger than $N_{class}$, since classification is based on the output probability matrix $P_{Y|X_{out}}$.

For a number of features $D=2^{d}$, the number of layers is *d*. If *D* is not a power of 2, then it is possible to use combiners with 3 or more inputs (the changes in the equations in Section 2.3 are straightforward, since a combiner with three inputs can be seen as two cascaded combiners with two inputs each).

The overall binary topology proposed in Figure 4 requires a number of information nodes equal to

(25) $$N_{nodes}=D+\frac{D}{2}+\frac{D}{4}+\cdots +2+1=2D-1$$

and a number of combiners equal to

(26) $$N_{comb}=\frac{D}{2}+\frac{D}{4}+\cdots +2+1=D-1$$

All the info nodes run exactly the same algorithm and all the combiners are equal, apart from the input/output alphabet cardinalities. If the cardinalities of the alphabets are all equal, i.e., $N_{in}^{\left(i\right)}$ and $N_{out}^{\left(i\right)}$ do not depend on the layer *i*, then all the nodes and all the combiners are exactly equal, which might help in a possible hardware implementation; in this case, the number of parameters of the network is $\left(N_{out}-1\right)\times N_{in}\times N_{nodes}$.

Actually, the network performance depends on how the features are coupled in subsequent layers and a random shuffling of the columns of matrix $X_{train}$ provides results that might be significantly different. This property is used in Section 3.1 for building the ensemble of networks.

### 2.5. A Note on Computational Complexity and Memory Requirements

The modular structure of the proposed method has several advantages in terms of both memory footprint and computational cost. The considered topology in this explanation is binary, similarly to what is depicted in Figure 4. We furthermore consider for simplicity cardinalities of the *D* input features all equal to $N_{in}$ and input/output cardinalities of subsequent layers information node to also be fixed constants $N_{in}^{*}$ and $N_{out}^{*}=\frac{N_{in}^{*}}{2}$, respectively. As we show in the experiment (Section 5), small values for $N_{in}^{*}$ and $N_{out}^{*}$ such as 2, 3, or 4 are sufficient in the considered cases. Straightforward generalizations are possible when considering inhomogeneous cases.

At the first layer (the input node layer), each of the *D* input nodes stores the joint probabilities of the target variable *Y* and its input feature. Each node thus includes a simple counter that fills the probability matrix of dimension $N_{in}\times N_{class}$. Both the computational cost and the memory requirements for this first stage are the same as the Naive Bayes algorithm. Notice that, from the memory requirements point of view, it is not necessary to store all the training data but just counters with number of joint occurrences of features/classes. If after training, new data are observed, it is in fact sufficient to update the counters and properly renormalize the values to obtain the updated probability matrices. In this paper, we do not cover the topic of online learning as well as possible strategies to reduce the computational complexity in such a scenario.

At the second layer (the first information node layer), each node receives as input the joint probability matrix of feature and target variable and performs the Blahut–Arimoto algorithm. The internal memory requirement of this node is the space needed to store two probability matrices of dimensions $N_{in}^{*}\times N_{class}$ and $N_{in}^{*}\times N_{out}^{*}$, respectively. The cost per iteration of Blahut–Aritmoto depends on matrix multiplication of sizes $N_{in}^{*}\times N_{out}^{*}$ and $N_{in}^{*}\times N_{class}$, and thus obviously the complexity scales with the number of classes of the considered classification problem. To the best of our knowledge, the convergence rate for the Blahut–Arimoto algorithm applied to information bottleneck problems is unknown. In this study, however, we found empirically that, for the considered datasets, 5–6 iterations per node are sufficient, as discussed in Section 5.5.

Each combiner process the matrices generated by two information nodes: the memory requirement is zero and the computational cost is roughly $N_{class}$ Kronecker products between rows of probability matrices. Since for ease of explanation we chose $N_{out}^{*}=\frac{N_{in}^{*}}{2}$ the output probability matrix have again dimensions $N_{in}^{*}\times N_{class}$.

The overall memory requirement and computational complexity (for a single DIN) is thus going to scale as *D* times the requirements for an input node, 2D−1 times the requirements for an information node, and D−1 times the requirements for a combiner. To complete the discussion, we have to remember that a further multiplication factor of $N_{mach}$ is required to take into account that we are considering an ensemble of networks (actually, at the first layer, the set of input nodes can be shared by the different architectures since only the relative position of the input nodes changes, see Section 3.1).

## 3. The Running Phase

During the running phase, the columns of matrix X with *N* rows and *D* columns are used as inputs. Assume again that the network architecture is that depicted in Figure 4 with D=8, and consider the *n*th input row X(n,:).

In particular, assume that X(n,2k)=i and X(n,2k+1)=j. Then,

  (a) input node I(2k) passes value *i* to info node (2k,0);
  (b) input node I(2k+1) passes value *j* to info node (2k+1,0);
  (a) info node (2k,0) passes the probability vector $p_{a}=P_{X_{out}\left(2k,0\right)|X_{in}\left(2k,0\right)}\left(i,:\right)$ (*i*th row) to the combiner; $p_{a}$ stores the conditional probabilities $P(X_{out}\left(2k,0\right)=g|X\left(n,2k\right)=i)$ for $g=0,\ldots ,N_{out}^{\left(0\right)}-1$;
  (b) info node (2k+1,0) passes the probability vector $p_{b}=P_{X_{out}\left(2k+1,0\right)|X_{in}\left(2k+1,0\right)}\left(j,:\right)$ (*j*th row) to the combiner; $p_{b}$ stores the conditional probabilities $P(X_{out}\left(2k+1,0\right)=h|X\left(n,2k+1\right)=j)$ for $h=0,\ldots ,N_{out}^{\left(0\right)}-1$;
the combiner generates vector
  (27) $$p_{c}=p_{a}⊗p_{b},$$
  which stores the conditional probabilities $P(X_{in}\left(k,1\right)=s|X\left(n,2k\right)=i,X\left(n,2k+1\right)=j)$ for $s=0,\ldots ,N_{in}^{\left(1\right)}-1$, where $N_{in}^{\left(1\right)}=N_{out}^{\left(0\right)}\times N_{out}^{\left(0\right)}$;
info node (k,1) generates the probability vector
  (28) $$p_{c}P_{X_{out}\left(k,1\right)|X_{in}\left(k,1\right)},$$
  which stores the conditional probabilities $P(X_{out}\left(k,1\right)=r|X\left(n,2k\right)=i,X\left(n,2k+1\right)=j)$ for $r=0,\ldots ,N_{out}^{\left(1\right)}$
in the following layer, each combiner performs the Kronecker product of its two input vectors and each info node performs the product between the input vector and its conditional probability matrix $P_{X_{out}|X_{in}}$;
the root information node at Layer 3, having the input vector p, outputs
  (29) $$p_{out}\left(n\right)=pP_{X_{out}\left(0,3\right)|X_{in}\left(0,3\right)}P_{Y|X_{out}\left(0,3\right)},$$
  which stores the estimated probabilities P(Y=m|X(n,:)) for $m=0,\ldots ,N_{class}-1$.
  According to the MAP criterion, the estimated class of the input point X(n,:) is
  (30) $$\hat{Y}\left(n\right)=\arg\max p_{out}\left(n\right)$$
  but we propose to use an improved method, as described in Section 3.1.

### 3.1. The DIN Ensemble

At the end of the training phase, when all the conditional matrices have been generated in each information node and combiner, the network is run with input matrix $X_{train}$ ($N_{train}$ rows and *D* columns) and the probability vector $p_{out}$ is obtained for each input point $X_{train}\left(n,:\right)$. As anticipated at the end of Section 2.4, the DIN classification accuracy depends on how the input features are combined together. By permuting the columns of $X_{train}$, a different probability vector $p_{out}$ is typically obtained. We thus propose to generate an ensemble of DINs by randomly permuting the columns of $X_{train}$, and then combine their outputs.

Since in the training phase y(n) is known, it is possible to get for each DIN *v* the probability $p_{out}^{v}\left(n\right)$, and ideally $p_{out}^{v}\left(n,y\left(n\right)\right)$, the estimated probability corresponding to the true class y(n), should be equal to one. The weights

(31) $$w^{v}=\frac{\sum_{n=0}^{N_{train}-1}p_{out}^{v}\left(n,y\left(n\right)\right)}{\sum_{n=0}^{N_{train}-1}\sum_{j=0}^{N_{mach}-1}p_{out}^{j}\left(n,y\left(n\right)\right)}$$

thus represent the reliability of the *v*th DIN.

In the running phase, feeding the $N_{mach}$ machines each with the correctly permuted vector X(n,:), the final estimated probability vector is determined as

(32) $${\hat{p}}_{ens}\left(n\right)=\sum_{v=0}^{N_{mach}-1}w^{v}{\hat{p}}_{out}^{v}\left(n\right)$$

and the estimated class is

(33) $$\hat{Y}\left(n\right)=\arg\max{\hat{p}}_{ens}\left(n\right).$$

## 4. The Probabilistic Point of View

This section is intended to underline the difference in probability terms formulation between the Naive Bayes classifier [2,26] and the proposed scheme, since both use the assumption of conditional independence of the input features. Both classifiers build in a simplified way the probability matrix $P_{Y|X_{0},\ldots ,X_{D}}$ with $N_{class}$ rows and $\prod_{i=0}^{D-1}N_{in}^{\left(i\right)}$, where $N_{in}^{\left(i\right)}$ is the cardinality for the input feature $X_{i}$. In the next sections, we show the different structure of these two probability matrices.

### 4.1. Assumption of Conditionally Independent Features

The Naive Bayes assumption allows writing the output estimated probability of the Naive Bayes classifier as follows:

(34) $$\begin{array}{cc} P(Y=m|x=x_{0})\; & =\frac{P\left(x=x_{0}|Y=m\right)P\left(Y=m\right)}{P(x=x_{0})}\\ \; & =\frac{\left[\prod_{k=0}^{D-1}P\left(X_{k}=x_{k0}|Y=m\right)\right]P\left(Y=m\right)}{\sum_{s=0}^{N_{class}}\left[\prod_{k=0}^{D-1}P\left(X_{k}=x_{k0}|Y=s\right)\right]P\left(Y=s\right)} \end{array}$$

which is very easily implemented, without the need of generating the tree network. We rewrite this output probability in a fairly complex way to show the difference between the naive Bayes probability matrix and the DIN one. Consider the *n*th feature x(n), which can take values in the set $\left\{c_{n}^{0},\cdots ,c_{n}^{D_{n}-1}\right\}$. Define $p_{x(n)|y=m}=\left[P\left(x\left(n\right)=c_{n}^{0}|Y=m\right),\cdots P\left(x\left(n\right)=c_{n}^{D_{n}-1}|Y=m\right)\right]$; then,

(35) $$P_{X_{in}|Y}\left(m,:\right)={⊗}_{k=0}^{D-1}p_{x(k)|y=m}$$

and thus obviously

(36) $$P_{X_{in}|Y}=\left[\begin{array}{c} {⊗}_{k=0}^{D-1}p_{x(k)|y=0}\\ {⊗}_{k=0}^{D-1}p_{x(k)|y=1}\\ \vdots \\ {⊗}_{k=0}^{D-1}p_{x\left(k\right)|y=N_{class}} \end{array}\right]$$

We can write the joint probability matrix as

(37) $$P_{X_{in},Y}=diag\left(P_{Y}\right)P_{X|Y}$$

and the probability matrix of target class given observation as

(38) $$P_{Y|X_{in}}={\left(P_{X_{in},Y}diag\left(P_{X_{in}}^{\circ (-1)}\right)\right)}^{T}$$

The hypothesis of conditional statistical independence of the features is not always correct and thus we can incur obvious performance degradation.

### 4.2. The Overall Probability Matrix

We now instead compute the output estimated probability for the DIN classifier. Consider again the sub-network in Figure 3 made of info nodes *a*, *b*, and *c*. Info node *a* is characterized by matrix $P_{a}$, whose element $P_{a}\left(i,j\right)$ is $P(X_{out,a}=j|X_{in,a}=i)$; similar definitions hold for $P_{b}$ and $P_{c}$. Note that $P_{a}$ and $P_{b}$ have $N_{0}$ rows and $N_{1}$ columns, whereas $P_{c}$ has $N_{1}\times N_{1}$ rows and $N_{2}$ columns; the overall probability matrix between the inputs $X_{in,a}$, $X_{in,b}$ and the output $X_{out,c}$ is $\tilde{P}$ with $N_{0}\times N_{0}$ rows and $N_{2}$ columns. Then,

(39) $$\begin{array}{cc} \; & P(X_{out,c}=i|X_{in,a}=j,X_{in,b}=k)\\ \; & =\sum_{r=0}^{N_{1}-1}\sum_{s=0}^{N_{1}-1}P\left(X_{out,c}=i,X_{out,a}=r,X_{out,b}=s|X_{in,a}=j,X_{in,b}=k\right)\\ \; & =\sum_{r=0}^{N_{1}-1}\sum_{s=0}^{N_{1}-1}P\left(X_{out,c}=i|X_{out,a}=r,X_{out,b}=s\right)P\left(X_{out,a}=r|X_{in,a}=j\right)P\left(X_{out,b}=s|X_{in,b}=k\right)\\ \; & =\sum_{r=0}^{N_{1}-1}\sum_{s=0}^{N_{1}-1}P\left(X_{out,c}=i|X_{out,s}=r,X_{out,b}=s\right)P_{a}\left(j,r\right)P_{b}\left(k,s\right). \end{array}$$

It can be shown that

(40) $$\tilde{P}=\left(P_{a}⊗P_{b}\right)P_{c}$$

where ⊗ identifies the Kronecker matrix multiplication; note that $P_{a}⊗P_{b}$ has $N_{0}\times N_{0}$ rows and $N_{1}\times N_{1}$ columns. By iteratively applying the above rule, we can get the expression of the overall matrix $\tilde{P}$ for the exact topology of Figure 4, with eight input nodes and four layers:

(41) $$\begin{array}{cc} \tilde{P} & =[\left\{\left[\left(P_{0,0}⊗P_{1,0}\right)P_{0,1}\right]⊗\left[\left(P_{2,0}⊗P_{3,0}\right)P_{1,1}\right]\right\}P_{0,2}\\ \; & ⊗\left\{\left[\left(P_{4,0}⊗P_{5,0}\right)P_{2,1}\right]⊗\left[\left(P_{6,0}⊗P_{7,0}\right)P_{3,1}\right]\right\}P_{1,2}]P_{0,3}. \end{array}$$

The overall output probability matrix $P_{Y|X}$ can finally be computed as

(42) $$P_{Y|X_{in}}=\tilde{P}P_{Y|X_{out}\left(0,3\right)}.$$

The DIN then behaves as a one-layer system that generates the output according to matrix $P_{Y|X_{in}}$, whose size might be impractically large. It is also possible to interpret the system as a sophisticated way of factorizing and approximating the exponentially large true probability matrix. In fact, the proposed layered structure needs smaller probability matrices, which makes the system computationally efficient. The equivalent probability matrix is thus different in the DIN (Equation (42)) and Naive Bayes (Equation (38)) cases.

## 5. Experiments

In this section, we analyze the results obtained with benchmark datasets. In particular, we consider the DIN ensemble when: (a) each DIN is based on the probability matrices (the scheme described in this paper); and (b) each information node of the DIN randomly generates the symbols, as described in the previous work [16]. We refer to these two variants in captions and labels as DIN(Prob) and DIN(Gen), respectively. The reason for this comparison is that conditional statistical independence is not required in the case DIN(Gen), and the classification accuracy could be different in the two cases. Note that Franzese and Visintin [16] considered just one DIN, not an ensemble of DINs. In the following, we introduce three datasets on which we tested the method (Section 5.1, Section 5.2 and Section 5.3) and propose some examples of DINs architectures. Complete analysis of numerical results is described in Section 5.4. Section 5.5 and Section 5.6 analyze the impact of changing the maximum number of iterations of Blahut–Arimoto algorithm and Lagrangian coefficient β, respectively. Finally, a synthetic multiclass experiment is described in Section 5.7. In all experiments, the value of β was optimized similarly to what is described in Section 5.6 using the training set.

### 5.1. UCI Congressional Voting Records Dataset

The first experiment on real data was conducted on the UCI Congressional Voting Records dataset [27], which collects the votes given by each of the U.S. House of Representatives Congressmen on 16 key laws (in 1985). Each vote can take three values corresponding to (roughly, see [27] for more details) yes, no, and missing value; each datum belongs to one of two classes (Democrats or Republican). The aim of the network is, given the list of 16 votes, decide if the voter is Republican or Democratic. In this dataset, we thus have D=16 features and 435 data split into $N_{train}$ data for training and $N_{test}=435-N_{train}$ data for testing. The architecture of the used network is the same as the one described in Section 2.4, except for the fact that there are 16 input features instead of 8 (the network has thus one more layer). The input cardinality in the first layer is $N_{in}^{\left(0\right)}=3$ (yes/no/missing) and the output cardinality is set to $N_{out}^{\left(0\right)}=2$. From the second layer on, the input cardinality for each information node is equal to $N_{in}^{*}=4$ and $N_{out}^{*}=2$. In the majority of the cases, the size of the probability matrices is therefore 4×2 or 2×2. In this example, we used $N_{mach}=30$ and $N_{train}=218$ (roughly 50% of the data). The value of β was set to 2.2.

### 5.2. UCI Kidney Disease Dataset

The second considered dataset was the UCI Kidney Disease dataset [28]. The dataset has a total of 24 medical features, consisting of mixed categorical, integer, and real values, with missing values. Quantization of non-categorical features of the dataset was performed according to the thresholds in Appendix A, agreed upon by a medical doctor.

The aim of the experiment is to correctly classify patients affected by chronic kidney disease. We performed 100 different trials training the algorithms using only $N_{train}=50$ out of 400 samples for the training. Layer zero has 24 input nodes, and then the outputs of layer zero are mixed two at a time to get 12 information nodes at Layer 1, 6 at Layer 2, and 3 at Layer 3; the last three nodes are combined into a unique final node. The output cardinality of all nodes is equal to $N_{out}^{*}=2$. The value of β was set equal to 5.6. In addition, in this case, we used an ensemble of $N_{mach}=30$ DINs.

### 5.3. UCI Mushroom Dataset

The last considered dataset was the UCI Mushroom dataset [29]. This dataset is comprised of 22 categorical features with different cardinalities, which describe some properties of mushrooms, and one target variable that defines whether the considered mushroom is edible or poisonous/unsafe. There are 8124 entries in the dataset. We padded the dataset with two null features to reach the cardinality of 24 and used exactly the same architecture as the kidney disease experiment. We selected $N_{train}=50$, β=2.7, and number of DINs equal to $N_{mach}=15$.

### 5.4. Misclassification Probability Analysis

We hereafter report results in terms of misclassification probability between the proposed method and several classification methods implemented using MATLAB^®^ Classification Learner. All datasets were randomly split 100 times into training and testing subsets, thus generating 100 different experiments. The proposed method shows competitive results in the considered cases, as can be observed in Table 1. It is interesting to compare in terms of performance the proposed algorithm with respect to the Naive Bayes classifier, i.e., Equation (34), and the Bagged Tree algorithm, which is the closest algorithm (conceptually) to the one we propose. In general, the two variants of the DINs perform similarly to the Bagged Trees, while outperforming Naive Bayes. For Bagged Trees and KNN-Ensemble, the same number of learners as DIN ensembles were used.

### 5.5. The Impact of Number of Iterations of Blahut–Arimoto on The Performance

**Figure 5:**
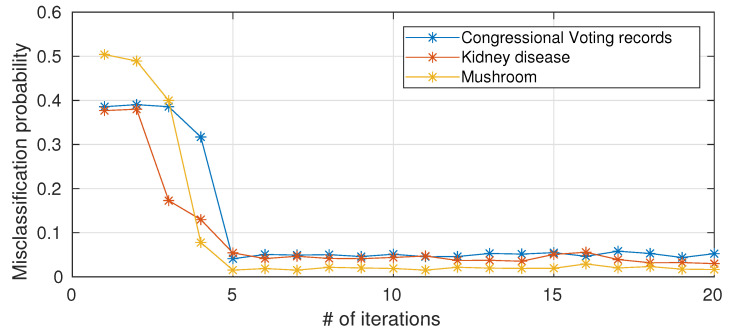
Misclassification probability versus number of iterations (average over 10 different trials) for the considered UCI datasets.

As anticipated in Section 2.5, the computational complexity of a single node scales with the number of iterations of Blahut–Arimoto algorithm. To the best of our knowledge, a provable convergence rate for the Blahut–Arimoto algorithm in the information nottleneck setting does not exist. We hereafter (Figure 5) present empirical results on the impact of limiting the number of iterations of Blahut–Arimoto algorithm (for simplicity, the same bound is applied to all nodes in the networks). When the number of iterations is too small, there is a drastic decrease in performance because the probability matrices in the information nodes have not yet converged, while 5–6 iterations are sufficient and a further increase in the number of iterations is not necessary in terms of performance improvements.

### 5.6. The Role of β: Underfitting, Optimality, and Overfitting

**Figure 6:**
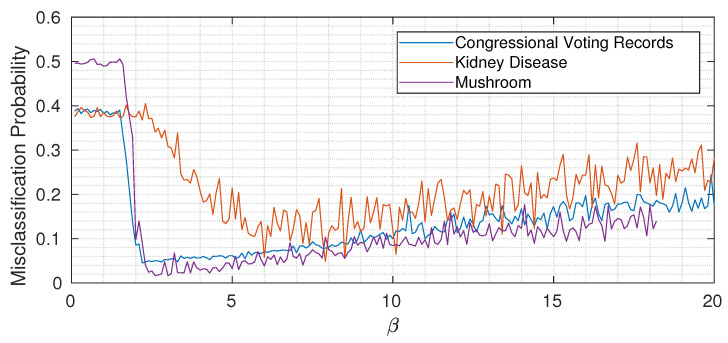
Misclassification probability versus β (average over 20 different trials) for the considered UCI datasets.

As usual with almost all machine learning algorithms, the choice of hyperparameters is of fundamental importance. For simplicity, in all experiments described in the previous sections, we kept the value of β constant through the network. To gain some intuition, Figure 6 shows the misclassification probability for different β for the three considered datasets (each time keeping β constant through the network). While the three curves are quantitatively different, we can notice the same qualitative trend: when β is too small, not enough information about the target variable is propagated, and then by increasing β above a certain threshold, the misclassification probability drops. Increasing β too much however induces overfitting, as expected, and the classification error (slowly) increases again. Remember (from Equation (15)) that the Lagrangian we are minimizing is

$$L=I\left(X_{in};X_{out}\right)-\beta I\left(Y;X_{out}\right).$$

Information theory tells us that at every information node we should propagate only the sufficient statistic about the target variable *Y*. In practice, this is reflected in the role of β: when it is too small, we neglect the term $I(Y;X_{out})$ and just minimize $I(X_{in};X_{out})$ (that corresponds to underfitting), while increasing β allows passing more information about the target variable through the bottleneck. It is important to remember, however, that we do not have direct access to the true mutual information values but just to an empirical estimate based on a finite dataset. Especially when the cardinalities of inputs and outputs are high, this translates into an increased probability of spotting spurious correlations that, if learned by the nodes, induce overfitting. The overall message is that β has an extremely important role in the proposed method, and its value should be chosen to modulate between underfitting and overfitting.

### 5.7. A Synthetic Multiclass Experiment

**Figure 7:**
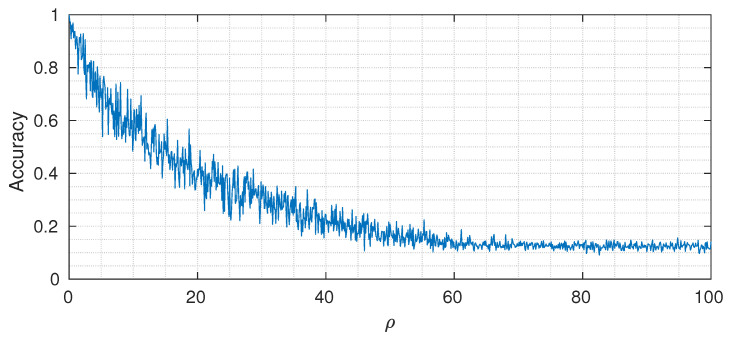
Varying of classification accuracy for different values of control parameter ρ.

In this section we present results on a multiclass synthetic dataset. We generated 64-dimensional feature vectors z drawn from multivariate Gaussian distributions with mean and covariance depending on a target class *y* and a control parameter ρ:

(43) $$\begin{array}{c} p\left(z|y=l\right)=|2\pi \Sigma_{l}{|}^{-\frac{1}{2}}\exp\left(-\frac{1}{2}{\left(z-\mu_{l}\right)}^{T}{\left(\rho \Sigma_{l}\right)}^{-1}\left(z-\mu_{l}\right)\right)\;l=1,\cdots ,N_{class} \end{array}$$

where for the considered experiment $N_{class}=8$. The mean $\mu_{l}$ is sampled from a normal 64-dimensional random vector and $\Sigma_{l}$ is randomly generated as $\Sigma_{l}=AA^{T}$ (where A is sampled from a matrix normal distribution) and normalized to have unit norm. The other parameter ρ is inserted to modulate the signal to noise ratio of the generated samples: a smaller value of ρ corresponds to smaller feature variances and more distinct, less overlapping, pdfs p(z|y=l), and an easier classification task. We then perform quantization of the result using 1 bit, i.e., the input of the ensemble of DINs is the following random vector:

(44) $$\begin{array}{c} x=U(z) \end{array}$$

where U(·) is the Heaviside step operator. The designed architecture has at the first layer 64 input nodes, followed by 32, 16, 4, 2, and 1. The output cardinalities are equal to 2 for the first three layers, 4 for the fourth and fifth layer, and 8 at the last layer. We selected $N_{train}=1000$, β=7 (constant trough the network), and number of DINs equal to $N_{mach}=10$. Figure 7 shows the classification accuracy (on a test set of 1000 samples) for different values of ρ. As expected, when the value of ρ is small, we can reach almost perfect classification accuracy, whereas, by increasing it, the performance drops to the point where the useful signal is completely buried in noise and the classification accuracy reaches the asymptotic level of $\frac{1}{8}$ (that corresponds to random guessing when the number of classes is equal to 8).

## 6. Conclusions

The proposed ensemble Deep Information Network (DIN) shows good results in terms of accuracy and represents a new simple, flexible, and modular structure. The required hyperparameters are the cardinality of the alphabet at the output of each information node, the value of the Lagrangian multiplier β, and the structure of the tree itself (number of input information nodes of each combiner).

Simplistic architecture choices made for the experiments (such as equal cardinality of all node outputs, β constant through the network, etc.) performed comparably to finely tuned networks. However, we expect that, similar to what happened in neural network applications, a domain specific design of the architectures will allow for consistent improvements in terms of performance on complex datasets.

Despite the local assumption of conditionally independent features, the proposed method always outperforms Naive Bayes. As discussed in Section 4, the induced equivalent probability matrix is different in the two cases. Intuitively, we can understand the difference in performance under the point of view of probability matrix factorization. On the one side, we have the true, exponentially large, joint probability matrix of all features and target class. On the other side, we have the Naive Bayes one, which is extremely simple in terms of complexity but obviously less performing. In between, we have the proposed method, where the complexity is still reasonable but the quality of the approximation is much better. The DIN(Gen) algorithm does not require the assumption of statistical independence, but the classification accuracy is very close to that of DIN(Prob), which further suggests that the assumption can be accepted from a practical point of view.

The proposed method leaves open the possibility of devising a custom hardware implementation. Differently from classical decision trees, in fact, the execution times of all branches as well as the precise number of operations is fixed per datum and known a priori, helping in various system design choices. In fact, with classical trees, where a node’s utilization depends on the datum, we are forced to design the system for the worst case, even if in the vast majority of time not all nodes are used. Instead, with DIN, there is no such a problem.

Finally, a clearly open point is related to the quantization procedure of continuous random variables. One possible self-consistent approach could be devising an information bottleneck based method (similar to the method for continuous random variables [20]).

Further studies on extremely large datasets will help understand principled ways of tuning hyperparameters and architecture choices and their relationship on performance. 

## Figures and Tables

**Table 1:** Mean misclassification probability (over 100 random experiments) for the three datasets with the considered classifiers.

Classifier	Congressional Voting Records	Kidney Disease	Mushroom
Naive Bayes	0.10894	0.051 g	0.20641
Decision Tree	0.050691	0.062314	0.05505
Bagged Trees	0.043641	0.0268	0.038305
DIN Prob	0.050138	0.037229	0.020796
DIN Gen	0.049447	0.026286	0.022182
Linear Discriminant Classifier	0.059724	0.091029	0.069923
Logistic Regression	0.075161	0.096429	0.07074
Linear SVM	0.063226	0.049914	0.04513
KNN	0.08682	0.11369	0.037018
KNN-Ensemble	0.062811	0.036057	0.043967

## Appendix A. Quantization

Hereafter, we present the quantization scheme used for the numerical features of chronic kidney disease dataset.

- Age (Years) {<10,<18,<45,<70,<120}
- Blood (mm/Hg) {<80,<84,<89,<99,<109,≥110}
- Blood Glucose Random (mg/dl) {<79,<160,<200,≥200}
- Blood Urea (mg/dl) {<6,<20,≥20}
- Serum Creatinine (mg/dl) {<0.5,<1.2,<2,≥2}
- Sodium (mEq/l) {<136,<145,≥145}
- Potassium (mEq/l) {<3.5,<5,≥5}
- Haemoglobin (gm) {<12,<17,≥17}
- Packed Cell Volume {<27,<52,≥52}
- White Blood Cell Count (cells/mm${}^{3}$) {<3500,<10500,≥10500}
- Red Blood Cell (millions/mm${}^{3}$) {<2.5,<6,≥6}
